# Using field size factors to characterize the in-air fluence of a proton machine with a range shifter

**DOI:** 10.1186/s13014-017-0783-2

**Published:** 2017-03-14

**Authors:** Jiajian Shen, Jarrod M. Lentz, Yanle Hu, Wei Liu, Danairis Hernandez Morales, Joshua B. Stoker, Martin Bues

**Affiliations:** Department of Radiation Oncology, Mayo Clinic Arizona, Phoenix, AZ USA

**Keywords:** Proton pencil beam scanning, Range shifter, Field size factor, Double Gaussians fluence, TPS commissioning

## Abstract

**Introduction:**

The range shifter (RS) is used to treat shallow tumors for a proton pencil beam scanning system (PBS). Adding RS certainly complicates the commissioning of the treatment planning system (TPS) because the spot sizes are significantly enlarged with RS. In this work, we present an efficient method to configure a commercial TPS for a PBS system with a fixed RS.

**Methods:**

By combining a spiral delivery with customized control points, we were able to significantly improve measurement efficiency and obtain 250 field size factors (FSF) within three hours. The measured FSFs were used to characterize the proton fluence and fit the parameters for the double-Gaussian fluence model used in the TPS. Extensive validation was performed using FSFs measured in air and in water, absolute doses of spread-out Bragg peak (SOBP) fields, and the dose measurements carried out for patient-specific quality assurance (QA).

**Results:**

The measured in-air FSFs agreed with the model’s prediction within 3% for all 250 FSFs, and within 2 for 94% of the FSFs. The agreement between model’s prediction and measurement was within 2% for the in-air and in-water FSFs and the absolute doses for SOBP beams. The patient-specific QA of 113 fields showed an excellent gamma passing rates (96.95 ± 2.51%) for the absolute dose comparisons with gamma criteria of 2 mm and 2%.

**Conclusion:**

The excellent agreement between the model’s prediction and measurements proved the efficiency and accuracy of the proposed method of using FSFs to characterize the proton fluence and configure the TPS for a PBS system with fixed RS.

## Introduction

The pencil beam scanning (PBS) technique has gained more popularity in proton centers [1] because PBS has reduced neutron dose, no need for apertures and compensators, and intrinsically enables intensity modulated proton therapy (IMPT) [2]. Due to technical challenges, current clinical PBS systems cannot provide protons with energies below 70 MeV [3]. This limits the ability to treat shallow tumors (<4 cm) unless a range shifter (RS) is added at the end of the nozzle.

The PROBEAT-V (Hitachi Ltd., Tokyo, Japan) proton system at our institution produces a nominal minimum energy beam of 71.3 MeV, corresponding to a range of 4 cm in water. A fixed RS with a water equivalent thickness (WET) of 4.5 cm can be attached at the end of the non-movable nozzle, 30 cm from isocenter. The dose algorithm used in our institution is the proton convolution superposition (PCS) dose algorithm in the Eclipse™ treatment planning system (TPS) (Varian Medical Systems, Palo Alto, CA). The PCS algorithm is a fluence-based dose model that calculates dose by convolving the proton fluence with a dose kernel hardcoded inside the TPS [4, 5].

The spot sizes of proton beamlets increase when the RS is used, and the increases are more pronounced for low proton energies [6]. This is due to the scattering of protons from the RS and the large air gap between the RS and isocenter. Additionally, the RS creates low-signal tails in the beam profile due to large-angle multiple Coulomb scattering and nuclear interactions. These low-signal tails can extend laterally more than 10 cm from the spot center [7, 8]. Clinically these low dose tails manifest themselves in an increase of output with field sizes [9], a phenomenon known as the field size effect. To address this clinically-relevant effect, the Eclipse PCS algorithm utilizes two Gaussian distributions to model the proton fluence: one for the primary fluence and the second for the tails [10]. The modeling process for a RS in a TPS is further complicated by spot size enlargement and the associated low-dose tails.

Methods for configuring the double-Gaussian fluence model in the Eclipse TPS have already been extensively investigated for a PBS system without a RS (vacuum machine) [10]. However, to the best of our knowledge no one has reported a method for configuring a TPS model for a PBS system with a RS. Since the primary spot sizes and tails caused by the RS are intrinsically quite different from those of the vacuum machine, the configuration of the model is different as well.

In this work, we propose an efficient method of using field size factor (FSF) to characterize the in-air proton fluence of a PBS system with a fixed RS, the results of which are then used to configure a double-Gaussian model in the Eclipse TPS. Throughout the paper, all beam energies refer to the nominal energies at the entrance of the nozzle.

## Methods and Materials

### Double-Gaussian model for proton fluence

The Eclipse PCS algorithm (Version 13.7.15) calculates dose by convolving the proton fluence with the proton dose kernel. The dose at a given point (x, y, z) may therefore be written as:

1 $$ D\left( x, y, z\right)={\displaystyle {\sum}_{E_k}}{\displaystyle {\sum}_{Kerne{ l}_j}}{\varnothing}_{E_k}\left( x, y, z;{x}_j,{y}_j, z\right){D}_{E_k}^{Kerne l}\left( x-{x}_j, y-{y}_j, d(z)\right) $$

where ${\varnothing}_{E_k}\left( x, y, z;{x}_j,{y}_j, z\right)$ is the proton fluence at position (x,y,z) caused by spot *j*, which is centered at (*x*_*j*_ *,y*_*j*_) and has energy *E*_*k*_, ${D}_{E_k}^{Kernel}\left( x-{x}_j, y-{y}_j, d(z)\right)$ is the dose kernel, d is the depth in medium, and z is the distance from isocenter along the beam direction. For the double-Gaussian fluence model, the distribution of protons is described as:

2 $$ {\varnothing}_{E_k}\left( x, y, z;{x}_j,{y}_j, z\right)={\varPhi}_{E_k}^j(z)\left[\frac{1-{w}_2\left({E}_k\right)}{2\pi {\sigma}_1^2\left({E}_k, z\right)} \exp \left(-\frac{{\left( x-{x}_j\right)}^2+{\left( y-{y}_j\right)}^2}{2{\sigma}_1^2\left({E}_k, z\right)}\right)+\frac{w_2\left({E}_k\right)}{2\pi {\sigma}_2^2\left({E}_k, z\right)} \exp \left(-\frac{{\left( x-{x}_j\right)}^2+{\left( y-{y}_j\right)}^2}{2{\sigma}_2^2\left({E}_k, z\right)}\right)\right] $$

where ${\varPhi}_{E_k}^j(z)$ is the maximum fluence of the spot *j*, *w*_2_(*E*_*k*_) is the weight of the second Gaussian function, and *σ*_1_(*E*_*k*_, *z*) and *σ*_2_(*E*_*k*_, *z*) are the standard deviations of the first and second Gaussian, respectively. Properly configuring the model is therefore a matter of finding the optimal values of *σ*_1_(*E*_*k*_, *z*), *σ*_2_(*E*_*k*_, *z*) and *w*_2_(*E*_*k*_).

### Field size factor measurements

**Fig. 1:**
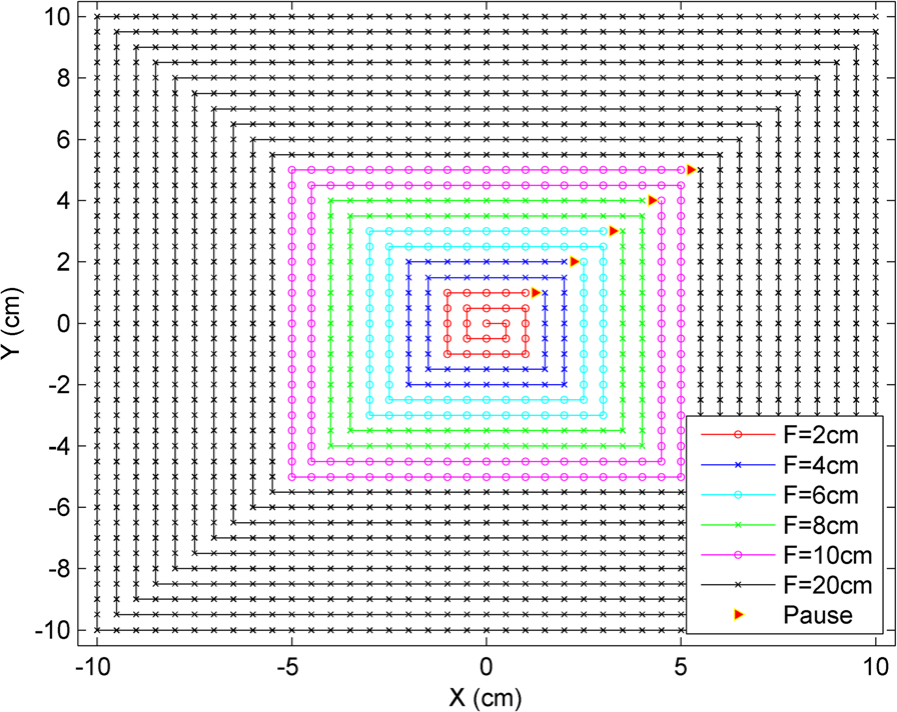
Spiral delivery pattern with control points for efficient FSF measurements. The red triangles show the control points used to pause the beam delivery and record the charge. Using the spiral pattern and the control points, a set of FSFs can be obtained by delivering just one beam with the maximum field size (i.e., 20 cm × 20 cm)

Single-energy spot arrays with a uniform spot spacing of 5 mm and a 20 cm square field size were generated for ten proton energies, ranging from 80.3 MeV to 175.9 MeV. The spot delivery pattern was designed in such a way that the proton beamlets were delivered in a spiral pattern, beginning at the center and proceeding outward. Five control points were also added in key places to create brief pauses in the beam delivery. The purpose of control points is usually to pause the delivery while the system switches from one proton energy to the next, an automatic process in out PTB system which takes approximately two seconds. In this case, however, they were used to provide time to manually pause the beam and record the charge collected by the ionization chamber before resuming delivery. The five control points were chosen so that the beam was paused after delivering field sizes of 2, 4, 6, 8, and 10 cm, as shown in Fig. 1. This allowed charge readings to be recorded for six field sizes (including the full 20 cm field) in around three minutes using a single beam delivery. Reloading the spot pattern files and waiting for the proton system’s various interlocks to clear takes time, so pausing one beam five times represents a significant improvement in efficiency over running six separate beams for six different field sizes.

In-air charge measurements were made at five positions along the beam direction (in the isocenter plane, ±10 cm and ±20 cm from isocenter) for ten proton energies ranging from 80.3 MeV to 175.9 MeV using six field sizes (2, 4, 6, 8, 10, and 20 cm). The measurements were made with a 34045 Advanced Markus ion chamber (PTW, Freiburg, Germany), which has an effective volume of 0.02 cm^3^, radius 2.5 mm and a 1.06 mm WET entrance window. To keep the chamber in place, it was inserted into a cutout within a 30 cm × 30 cm × 3 cm acrylic plate machined to hold the chamber. The chamber surface and the effective measurement point (1 mm below the surface) were aligned with the laser, and the couch was moved vertically to bring the chamber to the five measurement positions. At each new couch position, beam with the smallest field size (2 cm) was delivered five times at the center and at offsets by ±1 mm in the x and y directions from the center, which ensures that the chamber was properly centered before beginning the FSF measurements. The smallest field size (2 cm) was delivered five times and charges were collected with beam offsets from the current center by ±1 mm in the x and y directions, respectively. The reproducibility of the FSF measurements was checked by repeating them multiple times on multiple days.

**Fig. 2:**
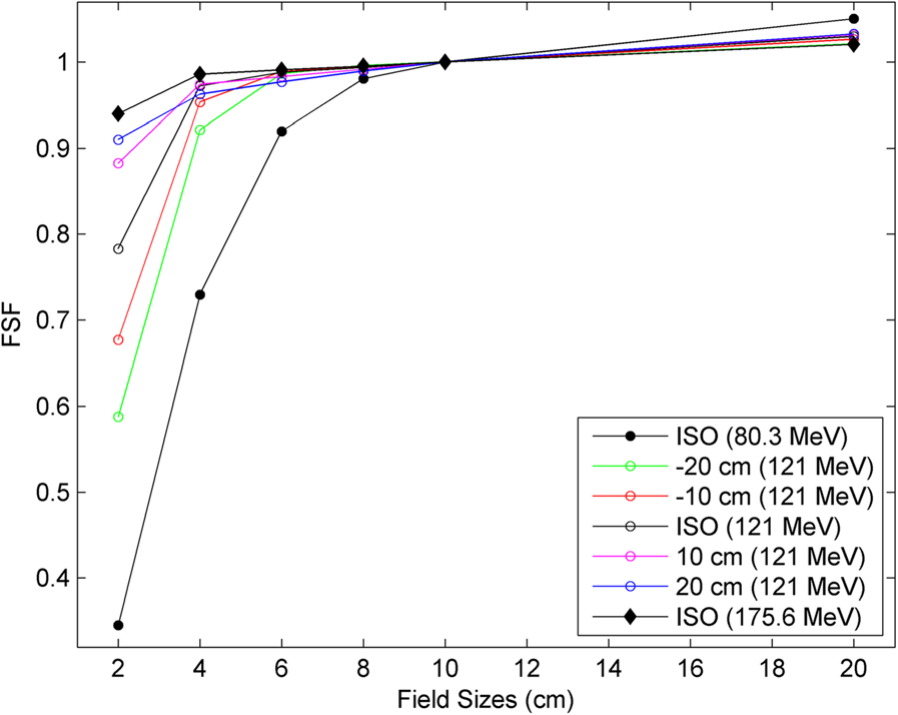
Measured FSFs at five positons (isocenter, ±10 cm and ±20 cm) for the proton energy of 121 MeV, and at the isocenter plane for the proton energies of 80.3 Mev and 175.6 MeV

The FSFs were obtained by normalizing the charges recorded at various field sizes to that of the 10 cm field. A total of 250 FSFs were measured and used to configure the proton fluence model (throughout this paper, the FSF for the 10 cm field was ignored, as it was 1.0 by definition). The FSFs for all five positions for 121 MeV protons are shown in Fig. 2, along with those for 80.3 MeV and 175.6 MeV protons at the isocenter position.

### Characterizing the parameters for proton fluence

The proton fluence distribution for a single spot was given previously in Eq. (2). The FSF for a given field size was derived by integrating the fluence distributions from all spots inside that field. The proton fluence (PF) at the center of the field for a given field size (FS) and spot spacing (SS) may be described as:

3 $$ P F\left( FS,\  SS\right)={\displaystyle \sum_{i=- N}^N}{\displaystyle \sum_{j=- N}^N}{\varnothing}_{spot}\left({r}_{i j}=\sqrt{{\left( i\times S{S}_x^{\hbox{'}}\right)}^2+{\left( j\times S{S}_y^{\hbox{'}}\right)}^2}\right) $$

Where $N=\frac{0.5\times FS}{S S},\kern0.5em  S{S}_x^{\hbox{'}}= S S\times \frac{ S S D+ d}{VSA{ D}_x},\ \mathrm{and}\kern0.5em  S{S}_y^{\hbox{'}}= S S\times \frac{ S S D+ d}{VSA{ D}_y}.$ *VSAD*_*x*_ and *VSAD*_*y*_ are the virtual SAD in the x and y directions, respectively. *SS*_*x*_^'^ and *SS*_*y*_^'^ are the projected spot spacing in the non-isocentric plane in the x and y directions, respectively. After the proton fluence was calculated for each square field, the FS were obtained by normalizing to the field size of 10 cm: $F S F=\frac{PF(FS)}{PF\left( FS=10\right)}$.

A large set of potential Gaussian fit parameters (*σ*_1_(*E*_*k*_, *z*), *σ*_2_(*E*_*k*_, *z*), and *w*_2_(*E*_*k*_)) were used to calculate FSFs for each energy at all five positions. The many calculated FSFs were then compared with the measured FSFs, and the optimal fit parameters were chosen to be those that led to the minimum differences between the measured and calculated FSFs.

With RS our proton beam therapy delivery system has a total of 68 energy layers between 80.3 MeV and 175.6 MeV. The optimal Gaussian fit parameters were chosen based on the ten energies that we measured, and the proton fluence parameters for the 58 remaining energies were interpolated based on these optimal fit parameters. All these fluence parameters were then put into the Eclipse TPS as part of the beam configuration process.

**Fig. 3:**
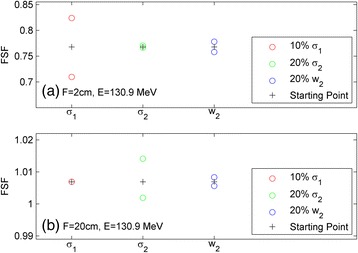
Sensitivity of the FSF to the proton spot fluence fit parameters for an energy of 130.9 MeV at field sizes of 2 cm (**a**) and 20 cm (**b**)

The sensitivity of the calculated FSFs to the various fit parameters was investigated and is demonstrated in Fig. 3. The ‘+’ symbol in the figure shows the initial calculated FSFs based on a typical set of fit parameters (*σ*_1_, *σ*_2_, and *w*_2_) for nominal energy E = 130.9 MeV. Each fit parameter was then adjusted separately and new FSFs were calculated. The red, green and blue circles in Fig. 3 show the new FSFs found by changing the fit parameters *σ*_1_, *σ*_2_, and *w*_2_ by ±10, ±20, and ±20%, respectively. The greater the change in the calculated FSF, the more sensitive the FSF is to that particular fit parameter. Figure 3a, for example, shows that the FSFs for FS = 2 cm are very sensitive to the primary Gaussian (*σ*_1_). A 10% primary Gaussian change causes >5% change in the FSFs for this smallest field size. However, the FSFs for this same FS = 2 cm are not sensitive to the secondary Gaussian at all: there was virtually no change in the calculated FSF when *σ*_2_ was changed by ±20%. Conversely, Fig. 3b shows that the FSFs for FS = 20 cm are not sensitive to the primary Gaussian but rather to the secondary Gaussian. The FSFs for both FS = 2 cm and 20 cm are mildly affected by the variation in the parameter *w*_2_. The data in Fig. 3 shows that with a set of FSFs from 2 cm to 20 cm, the double-Gaussian fluence parameters for a RS may be fitted with high accuracy because of these high sensitivities to the appropriate parameters in the respective field size ranges.

### Validation of the model

As was discussed previously, we measured the FSFs for ten energies to find the proton fluence parameters and used the results to interpolate the parameters for the remaining 58 energies. To verify that these interpolations were appropriate, the in-air FSFs were measured at the isocenter position for a different set of ten energies. A total of 50 FSFs were obtained to validate the interpolations.

Since all patient treatments involve the beams passing through a finite depth of tissue, the FSFs in water are clinically important. We measured 55 FSFs with various energies and depths in water and compared the results with the corresponding values obtained from the Eclipse TPS. This validated the model for single-energy FSFs in water.

FSFs are relative quantities. The absolute doses in the Eclipse TPS are from the integrated depth dose (IDD). In our commissioning process IDDs were generated by Monte Carlo simulation and normalized to the dose measured at a depth of 1 cm with a Bragg peak chamber. Various beams with different range values, spread-out Bragg peak (SOBP) widths, and field sizes were generated in the Eclipse TPS. These beams were delivered to a phantom using our proton beam delivery system the measured doses were compared with those from the Eclipse TPS. A small adjustment (up to 2%) to the IDD dose normalization was applied in order to achieve 2% dose agreement between the measured dose and the dose calculated by Eclipse TPS.

Dose planes for patient-specific QA were measured with the DigiPhant device (IBA Dosimetry, Schwarzenbruck, Germany) at multiple depths [11]. Measurements were also made with Gafchromic film with the one-dose protocol (Ashland, Bridgewater, NJ, USA) in order to take advantage of the high spatial resolution afforded by film measurements [12].

## Results

### Field size factors from best fit parameters

**Fig. 4:**
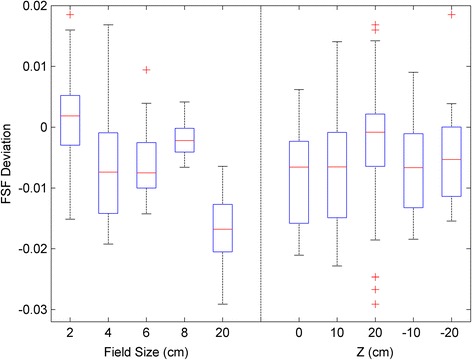
Differences in the FSF between the measurement and the calculation using optimal fit parameters. A total of 250 FSF comparisons are presented in two ways: grouped by the field size and by the distance from the isocenter. Each box plot represents a sample size of 50 FSF

Figure 4 shows the difference between measured FSFs and those calculated with the best fit parameters. A total of 250 data comparisons are presented in two ways: the left panel shows the differences grouped by various field sizes; while the right panel shows the differences grouped by the distances from isocenter. Each box plot corresponds to 50 data comparisons, and the minimum, first quartile, median, third quartile, maximum, and outliers are displayed in each box plot. Figure 4 shows that all calculated FSFs were within 3% from the measured FSFs, with most of them within 2%. Most of the FSFs with deviations greater than 2% correspond to the largest field size of 20 cm and those closest to the nozzle (i.e., 20 cm proximal to isocenter).

### Model validation results

**Fig. 5:**
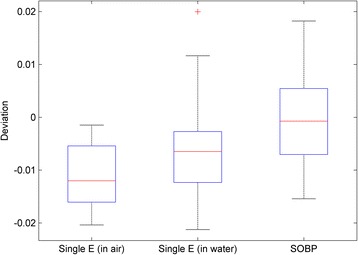
Validation of the model by the measurements. The first box plot has 50 in-air FSF comparisons for the ten new energies in which the calculated FSFs were obtained from the interpolated fluence parameters. The second box has 55 FSF comparisons for different energies and depths in water. The third box has 66 absolute dose comparisons between the calculations from the Eclipse TPS and the measurements for various ranges, SOBP widths, and field sizes

The results from the validation measurements discussed in section 2.4 are shown in Fig. 5. The first box plot shows the differences between the measured results and those calculated in the Eclipse TPS with the interpolated parameters for the 50 in-air single-energy FSFs. The second box plot shows the same for the 55 in-water single-energy FSFs. The third box plot shows the absolute dose difference between the Eclipse TPS and the measurements for the SOBP beams. All variations in the three box plots are within 2%, indicating that the model using parameters optimized based on FSFs is valid.

**Fig. 6:**
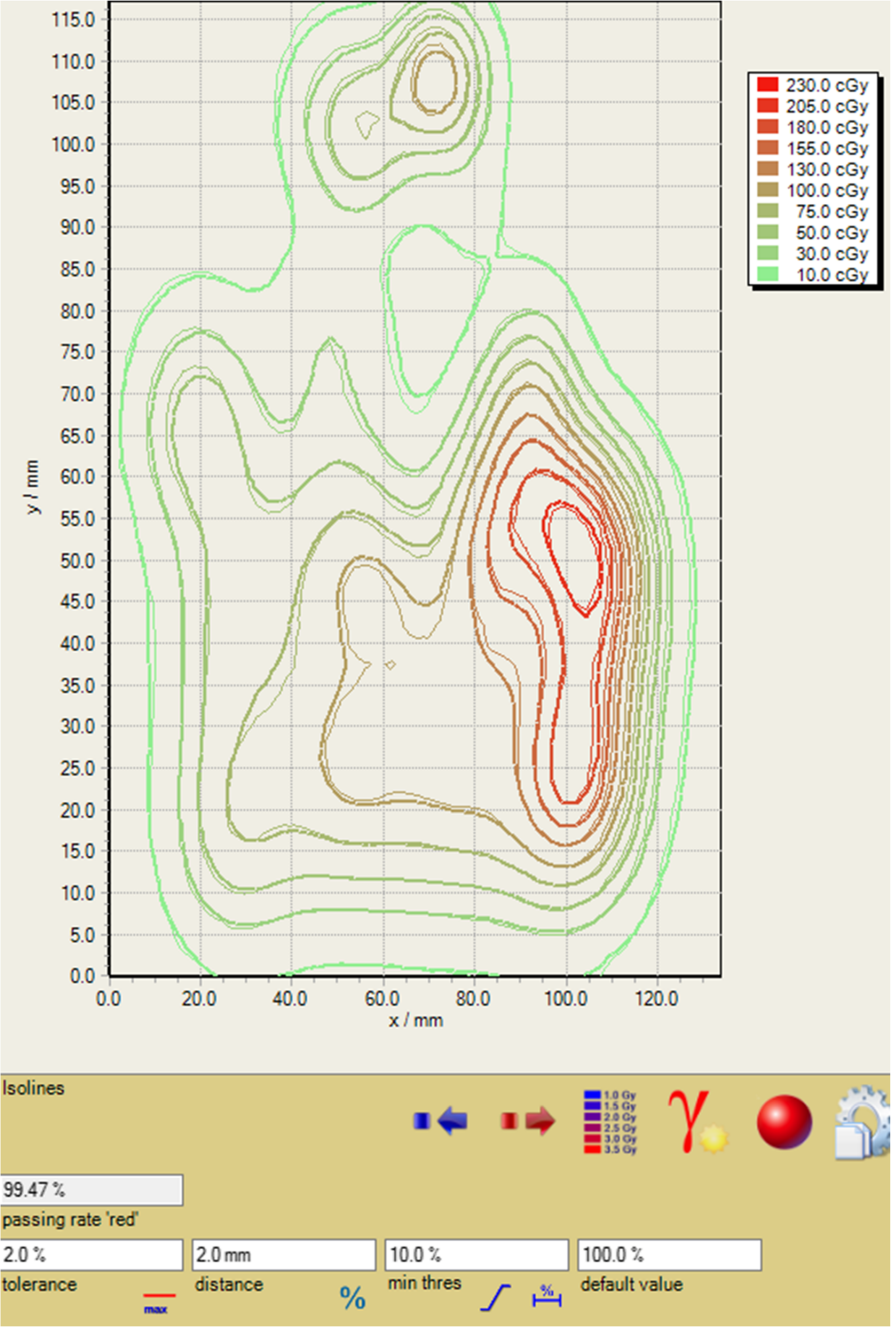
Patient-specific QA for an IMPT plan. The gamma passing rate for an absolute dose comparison was 99.47% for criteria of 2 mm, 2 and 10% threshold

Figure 6 shows the patient-specific QA result for an IMPT plan for a head-and-neck cancer patient. The measurements were made using Gafchromic film, and the gamma passing rate for the absolute dose comparisons with criteria of 2%, 2 mm, 10% threshold was 99.47%. The same kind of measurement (not shown here) was also done using a MatriXX PT, which gave a gamma passing rate of 99.36%. Overall, we have done patient-specific QA for 113 fields with the same criteria as mentioned above, and the gamma passing rates have been excellent (96.95% ±2.51%, ranging from 89.55 to 99.90%). These high gamma passing rates for patient QA solidifies the fact that the model with parameters fit by the FSFs is very good.

## Discussion

In this paper, we presented an efficient method for using FSFs to characterize the in-air proton fluence of single proton spots in the presence of a fixed RS. The derived proton fluence parameters for a double Gaussian model were used to configure the PCS algorithm in the Eclipse TPS. The method and the model were extensively validated by both in-air and in-water measurements for single-energy beams, SOBP beams, and patient-specific QA. All the validations show that the method is robust and the model is excellent.

The FSF measurements presented in this paper utilized a spiral delivery pattern and added control points. As shown in Fig. 1, this method is very efficient because several FSFs can be obtained by delivering a single beam. It took approximately three hours to measure 250 FSFs for ten energies at five different phantom positions relative to the proton nozzle. We then interpolated the optimal fit parameters for the proton fluence to the remaining energies. With these efficient FSF measurements, the entire proton fluence model was configured using direct measurements, without using any Monte Carlo simulation data. Instead, the FSF measurements provide useful information on in-air proton fluence that can improve the Monte Carlo simulation.

While the proton fluence for a single proton spot can be measured by high resolution detectors such as film, diodes, pinpoint chambers, and scintillators [7, 13], these measurements are usually time consuming, sensitive to setup errors, or need post-processing. It is especially challenging to measure the low-dose tails directly because a large number of monitor units are needed to increase the low-dose signals to the measurable level. Conversely, the FSFs were measured using homogeneous square fields, which are less affected by setup errors. The reproducibility tests for the repeated measurements on the same day and on different days showed a maximum deviation of < 0.5% and a standard deviation of 0.2%. Fig. 3a shows that the FSFs are very sensitive to the *σ*_1_ of the primary Gaussian for the 2 cm field size. For spot sizes less than 5 mm, it is quite possible that any direct spot profile measurements would bring sub-mm measurement uncertainties. However, Fig. 3a shows that even a 0.5 mm deviation may cause an error in FSFs greater than 5%, which is certainly not negligible. Therefore, the FSF measurements for the RS are more accurate and robust in characterizing the proton fluence.

Since IMPT has the capability to provide high dose conformity, it has increasingly been used in treating complex tumors, such as head-and-neck cancers [14]. For a highly-modulated plan, there are no homogenous dose distributions for any of the constituting fields, and the heavy modulation of each field generates high dose gradients inside the target. In cases such as this, it is especially critical to have an accurate model of both the primary proton fluence and that of the extended tails. If only a single-Gaussian model is used, the output can be off by more than 10%, as reported by Zhu et al. 2013 [10]. For the modelling of the extended tails, it is reported that a proper characterization of the tail component of the beam, below a factor of 10^−4^ of the central axis dose, is necessary to avoid dose inaccuracies [7]. Therefore, achieving an accurate dose calculation for an IMPT plan relies on the accurate modelling of every spot including 1^st^ and 2^nd^ Gaussian as well as their relative weights.

Figure 4 shows that the model with parameters derived from measured FSFs can predict FSFs to within 2% for most cases and to within 3% for all cases. It is worth noting that the proton spot profiles are not intrinsically double-Gaussian in shape, particularly for the laterally-extending low-dose tails. It was reported that adding an additional Cauchy-Lorentz function may fit measured profiles better [15]. Therefore, it is not unexpected that the double-Gaussian model does not fit every situation perfectly (e.g., within 2%). Nevertheless, as shown in Fig. 4, the few cases of up to 3% deviation were found for 20 cm field size and at a distance of 20 cm away from isocenter, both of which are clinically rare situations. Figure 5 shows that the accuracy of the fluence model was better than 2% for the FSFs measured in air and in water and for the absolute doses of the SOBP beams. These high-accuracy validations guarantee a precise model for the prediction of patient dose, as demonstrated by the high gamma passing rate for patient-specific QA shown in Fig. 6.

In this paper, we have limited the highest proton energy to 175.6 MeV, the residual range of which is 16.0 cm after the RS. Although this is deep enough for most of the cases that need a RS, it still possible that in some rare cases, such as that of a large tumor extending proximally to the body surface and distally deep in the body, higher proton energies may be needed. Measuring in-air FSFs is still an efficient way to accurately characterize the in-air proton fluence for energies higher than 175.6 MeV. However, our experience is that direct in-air proton fluence measurements for higher energies are not sufficient to configure the PCS algorithm in the Eclipse TPS. Firstly, with increasing energy there is more and more of the nuclear interactions that produce the low-dose tails (halo) in water [9]. The PCS algorithm in Eclipse has some deficiencies in modelling these halos, and we have to add some artificial proton fluence in air in order to compensate for these deficiencies [10]. Secondly, the higher energy protons have larger ranges in water. Thus, these protons undergo more scattering and the spot profiles change more as the beam travels through the water. The optimal fit parameters obtained from the in-air fluence remain valid only if the scattering model in the TPS is accurate and the fluence model in air matches the true fluence perfectly. Unfortunately neither of these ideal situations is reached in the current PCS algorithm in the Eclipse TPS. Therefore, as high energy protons go deep in the water, the optimal fit parameters found from the in-air measurements may not be the best fit for the spot profiles in water. It is probably necessary to measure the spot profiles in water and alter the in-air fluence model to match the in-water profiles for higher energies.

In our proton center, we have therefore used in-water FSFs for the proton energies higher than 175.6 MeV to configure the proton fluence in the TPS. By adding the artificial proton fluence, the above-mentioned two deficiencies were mitigated. Since the main purpose of this paper is to introduce an efficient method with direct in-air measurements to characterize the proton fluence, how to configure artificial proton fluence is beyond the scope of this paper. However, one can refer to a recent publication by Shen et al. 2016 for the details on configuring the artificial fluences [16].

## Conclusion

We presented an efficient method for FSF measurements utilizing a spiral spot delivery pattern and inserted control points. The measured in-air FSFs were used to fit a double-Gaussian fluence model to characterize the in-air proton fluence. The optimal fit parameters were used to configure the PCS algorithm in the Eclipse TPS. For TPS we have now reached an accuracy of 2% for in-water single-energy FSFs and for absolute dose of SOBP fields. We have also achieved a high gamma passing rate for patient-specific QA.
